# Development and validation of the comprehensive assessment scale for chemotherapy–induced peripheral neuropathy in survivors of cancer

**DOI:** 10.1186/s12885-019-6113-3

**Published:** 2019-09-10

**Authors:** K. Kanda, K. Fujimoto, R. Mochizuki, K. Ishida, B. Lee

**Affiliations:** 1 Department of Nursing, Takasaki University of Health and Welfare, 501 Nakaoruimachi, Takasakishi, Gunma 370-0033 Japan; 2 The Jikei University School of Medicine, School of Nursing, 8-3-1, Kokuryocho, Chofu, Tokyo 182-8570 Japan; 3 Niigata College of Nursing, 240 Shinnancho, Joetsu, Nigata 943-0147 Japan; 4 Department of Occupation, Gunma University Graduate School of Health Sciences, 3-39-22, Showamachi, Maebashi, Gunma 371-8514 Japan

**Keywords:** Cancer survivors, CIPN, Scale, PRO, Comprehensive assessment, Measurement, Symptom, QOL

## Abstract

**Background:**

Appropriate assessment is essential for the management of chemotherapy-induced peripheral neuropathy (CIPN), an intractable symptom that cannot yet be palliated, which is high on the list of causes of distress for cancer patients. However, objective assessment by medical staff makes it easy to underestimate the symptoms and effects of CIPN in cancer survivors. As a result, divergence from subjective evaluation of cancer survivors is a significant problem. Therefore, there is an urgent need to develop a subjective scale with high accuracy and applicability that reflects the experiences of cancer patients. We developed a comprehensive assessment scale for CIPN in cancer survivors, named the Comprehensive Assessment Scale for Chemotherapy-Induced Peripheral Neuropathy in Survivors of Cancer (CAS-CIPN), and demonstrated its reliability and validity.

**Methods:**

We developed a questionnaire based on qualitative studies of peripheral neuropathy in Japanese cancer patients and literature review. Twelve cancer experts confirmed the content validity of the questionnaire. A draft version comprising 40 items was finalized by a pilot test on 100 subjects. The participants in the present study were 327 Japanese cancer survivors. Construct validity was determined by factor analysis, and internal validity by confirmation factor analysis and Cronbach’s α.

**Results:**

Factor analysis showed that the structure consisted of 15 items in four dimensions: “*Threatened interference in daily life by negative feelings”*, “*Impaired hand fine motor skills”*, “*Confidence in choice of treatment/management,”* and “*Dysesthesia of the palms and soles.”* The CAS-CIPN internal consistency reliability was 0.826, and the reliability coefficient calculated using the Spearman-Brown formula [*q* = 2*r*/(1 + *r*)] was 0.713, confirming high internal consistency and stability. Scores on this scale were strongly correlated with Gynecologic Oncology Group-Neurotoxicity scores (*r* = 0.714, *p* < 0.01), confirming its criterion-related validity.

**Conclusions:**

The CAS-CIPN is an assessment tool with high reliability and validity for the comprehensive evaluation of CIPN in cancer survivors. The CAS-CIPN is simple to use, and can be used by medical professionals for appropriate situational assessment and intervention.

## Background

One in two people in Japan will develop cancer, and in 2018 the yearly number of new cases was projected to exceed 1 million [1, 2]. Cancer treatment must not only extend the survival, but also preserve the quality of life (QOL). From a survey of 4000 cancer patients undergoing outpatient chemotherapy, it was reported that chemotherapy-induced peripheral neuropathy (CIPN), an intractable symptom that cannot yet be palliated, was high on their list of causes of distress [3]. The main symptoms are numbness, pain, ache, and similar physical sensory disturbances on the hands and feet. Furthermore, it may also cause motor disturbances such as weakness and paralysis as well as hearing impairment. Its effects extend to restricting everyday activities, such as cooking and social roles, causing lifestyle breakdown [4, 5]. If the adverse effects become more severe, patients become vulnerable to falls [6, 7], burns, and driving errors, potentially imperiling their safety. Moreover, CIPN can lead to changes in the treatment schedule, such as the reduced doses or suspension of treatment, which may reduce the therapeutic effect [8]. Therefore, this affects survival as well as the assurance of safety and maintenance of QOL [8, 9]; resolving CIPN is thus an urgent task.

The reported incidence of CIPN in the literature varies widely from 10 to 100% [10]. Its incidence is high for platinum agents and taxanes, which are used in the treatment of lung, colorectal, and breast cancers, among the most common cancers worldwide [11]. A meta-analysis of 31 studies involving 4179 patients with colorectal, breast, or other cancers found that the timing of onset is within 1 month of the start of chemotherapy in 68.1% (57.7–78.4%) of cases, and ≥ 6 months in 30.0% (6.4–53.5%) [12]. CIPN, a serious symptom that presents from the start of treatment until > 12 months of treatment completion, imposes a heavy physical and mental burden on cancer survivors [13, 14]. In taxane-associated CIPN, mild symptoms usually improve with the reduction of the dose, but paclitaxel induced neuropathic pain and sensory abnormalities many persist for months or years after paclitaxel therapy [15]. Its appropriate management is thus extremely important, and the basis of management is an appropriate assessment of CIPN.

Many types of cancer chemotherapy are administered as outpatient treatments; however, the only drug therapy for which there is high-level evidence of palliative effect on numbness or painful symptoms is duloxetine [11]. A scale is thus required that is easy to use during the short time provided during outpatient appointments, and that appropriately assesses CIPN symptoms and their effects with high reliability and validity.

Although existing CIPN assessment tools include both objective and subjective tools, there is no generally used assessment tool based on widespread consensus [16–18]. In a systematic review, Griffith et al. [19] conducted a review of CIPN assessment tools published between 1980 and 2015; Haryani et al. [16] further developed on Griffith et al.’s work. Of the 20 tools surveyed, both studies identified two tools (FACT-GOG-Ntx, TNS) as recommended for use. Curcio also reviewed patients’ self-reported questionnaires, in investigating 7 scales described in 16 articles that met the set criteria for inclusion, but found no generally accepted assessment tool [17].

Common Terminology Criteria for Adverse Events (CTCAE) published by the National Cancer Institute is widely used in the field of therapeutic oncology worldwide. However, it has the problem of broad index categories, and findings by different evaluators may vary [18, 20]. In a comparison between medical professionals objective evaluations and patients subjective evaluations by patients, medical professionals tend to underestimate, resulting in disparity of assessments [21]. The emphasis has therefore now shifted to subjective patient-reported outcomes (PRO) [22].

The subjective tools used in Japan include the Japan-developed Patient Neurotoxicity Questionnaire (PNQ) [21] and self-check sheet [23]. Others include the Japanese versions of the Total Neuropathy Score (TNS) [24] and the Functional Assessment of Cancer Therapy/Gynecologic Oncology Group-Neurotoxicity (GOG-Ntx) [25]; both of which were developed overseas. The reliability and validity of neither the PNQ and self-check sheet nor the TNS were established at the time of their development. The TNS combines a patients’ subjective evaluation with medical professionals objective evaluationl; however, since training is required to administer the objective evaluation, its lack of versatility is a problem. As a subjective scale of demonstrated reliability and validity, the GOG-Ntx is a better scale [16, 19, 26].

However, there are two problems with these assessment tools. The first is that they do not reflect some actions that are an integral part of the Japanese lifestyle, such as chopstick use. This issue is not limited to Asian countries, as increasing internationalization means that it is now a problem worldwide. The second is that the effects of CIPN symptoms are not limited to activities of daily living, and these scales do not measure their general effect from the psychological, social, and spiritual perspectives. These problems impede an appropriate evaluation, meaning that symptoms may be underestimated.

The development of a versatile subjective scale capable of comprehensively measuring symptoms of persistent CIPN (PCIPN) and their effects, experienced by Japanese cancer survivors, is thus an urgent task. We developed a comprehensive assessment scale for measuring the effects of CIPN experienced by cancer survivors, and demonstrated its reliability and validity.

## Methods

### Terminology

#### Chemotherapy-induced peripheral neuropathy

This is a functional impairment of the sensory, motor, or autonomic nerves induced by cancer chemotherapy, and its resulting peripheral nervous signs or symptoms; and it is considered “persistent” if it lasts for more than 14 days.

#### Effects of CIPN

These are outcomes shown as signs or symptoms of CIPN that are recognized by cancer survivors as influencing them physically, psychologically (mentally), socially, or spiritually, or affecting their daily lives, as well as their response to these.

### Conceptual model of the scale under development

**Fig. 1:**
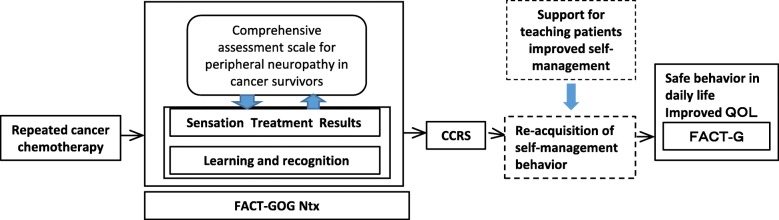
Model of the development of the comprehensive assessment scale for peripheral neuropathy in cancer survivors. CCRS: Chemotherapy Concerns Rating Scale, QOL: Quality of life, GOG NTx: Gynecologic Oncology Group-Neurotoxicity, FACT-G: Functional Assessment of Cancer Therapy-General

The scale developed in this study [named the Comprehensive Assessment Scale for Chemotherapy-induced Peripheral Neuropathy (CAS-CIPN) in Survivors of Cancer], is a comprehensive subjective assessment scale. The development model for this scale followed a symptom management conceptual model [27] and social cognitive theory [28], (Fig. 1). The experience of symptoms, symptom management strategies, and outcomes are all interrelated in the response to CIPN. Due to repeated treatments, survivors not only experience CIPN as physical symptoms but also recognize its effects on the social, mental/psychological, and spiritual terms as well as on their daily lives. Their response to these effects is a process that varies dynamically according to learning theory and how they perceive the world around them.

To make it comprehensive, this scale, which measures the experience of CIPN symptoms and their effects, was conceptualized using four subconcepts: physical sensations, effect on daily life, effect on relationships and social roles, and mental/psychological/spiritual effects. These were prioritized in the same order as the GOG-Ntx.

### Process of producing the initial version of the CAS-CIPN in survivors of cancer

#### Preliminary study to isolate constructs

To identify constructs, we investigated the experiences of 20 Japanese survivors with PCIPN. A total of 336 expressed experiences were recorded, which revealed that its effects did not only cause physical suffering but also social and mental distress and spiritual pain [29, 30]. We also reviewed the literature on the experience of CIPN and associated scales. The European Organization for Research and Treatment of Cancer developed a QOL questionnaire on CIPN (QLQ-CIPN20) [31], and although no Japanese version exists, this has been used in the USA and Canada as reported by Dolan et al. [32]. We also found another chronic scale developed for use with oxaliplatin, the Neurotoxicity Criteria of DEBIOPHARM (DEB-NTC) scale [33]. We referred to the PNQ, TNS, GOG-Ntx, DEB-NTC, CTCAE, and QLQ-CIPN20 in designating the four subconcepts for comprehensive assessment in this study.

#### Production of a draft version of the scale

For each of the four subconcepts, the codes obtained at the preliminary study were included in the item pool. Other items were added as a result of our review of the literature and in brainstorming sessions by the researchers. Duplications of semantic content and the simplicity of expression of each item were discussed between the researchers, and the items were repeatedly revised, resulting in a 112-item questionnaire (the draft version). The questions, which focused on CIPN symptoms and their effects, were preceded by the following text: “This questionnaire asks about the state of symptoms such as chronic (persistent) numbness continuing for 14 days or more, resulting from treatment drugs, and how these symptoms are affecting your daily life and feelings. Please mark the number that best applies to your condition during the past 7 days from 0 to 4” (items were evaluated on a five-point Likert scale from 0 to 4). 0 (strongly disagree) to 4 (strongly agree).

#### Investigation of content validity by cancer experts

Advice on the appropriateness of the draft version was obtained from 15 experts, comprising clinical nurse specialists (CNS) specializing in cancer nursing (including three CNS from Korea), four researchers (one of whom was also a clinical nurse specialist), and three cancer specialist doctors. The experts evaluated the content validity from three perspectives: (1) whether the questions expressed the symptoms of chronic peripheral neuropathy and their effects; (2) which effects of the 4 subconcepts were expressed by the questions; and (3) whether the questions conveyed their meaning and were easy to answer. Responses were obtained from 12 experts (7 CNS, 4 researchers [1 of whom was also a CNS], and 1 doctor; 80% response rate). In assessment (2) above, the agreement rate between the 112 questions and the subconcepts was 89.6%. Items that were difficult to answer due to age or sex, items that were indeterminable, and items that would have a major effect on subjects were considered; thus, 90 items were ultimately included in the draft version.

### Production of the final version, by correcting the draft version following the pilot test

A pilot test was conducted at two hospitals in eastern Japan, from October 2013 to June 2014, including patients who underwent at least 6 courses of regimens using Elplat or taxane (drugs that causes chronic peripheral neuropathy), and had continued on these therapies. Responses were received from 100 (87.5% response rate) men and women with colorectal, breast, or uterine cancers, and the distribution of responses to items was analyzed. Means ± standard deviations indicated the presence of ceiling and floor effects, items with effective content were isolated, and the final version of the scale consisting of 40 items was produced. Items reflecting Japanese culture, such as “chopstick use,” were excluded from the 40 items.

### Main study

#### Study participants and survey period

The study participants, who were patients from five hospitals in eastern Japan, had been diagnosed with cancer, and met the following criteria. (1) They underwent at least 6 courses of regimens using platinum or taxane (drugs that causes CIPN), and had continued on these therapies; (2) People who experienced paresthesia due to peripheral neuropathy in the hands and/or feet; (3) had performance status (PS) score 0–2; and (4) were in a stable mental and physical condition with sufficient cognitive and writing capacity to respond to daily conversation and questionnaires. The nurses introduced eligible study participants to the researchers, who conducted the study between August 2014 and January 2016. The sample size was set at 280 (40 questions × 7), based on the internal consistency criteria (‘Excellent’)for sample size (number of items × 7 and ≥ 100 subjects) in the COnsensus-based Standards for the selection of health Measurement INstruments (COSMIN) guidelines.

#### Questionnaire structure

In addition to the final version of the CAS-CIPN, questionnaires for the following existing scales were used to investigate participants’ attributes. It took from 15 to 20 min to complete the entire questionnaire.

(i) GOG-Ntx [25]: this is an additional subscale of FACT-G, an 11-question survey of the neurotoxicity of taxane-based chemotherapy drugs. Its reliability and validity have been demonstrated with Cronbach’s α 0.84–0.90. Each item is scored from 0 to 4 points to provide a total score out of 44, with higher scores indicating more severe neuropathic symptoms.
(ii) Functional Assessment of Cancer Therapy-General (FACT-G) (version 4) [34]: This scale measures cancer-specific health-related QOL, and the Cronbach’s α for the entire scale was 0.89. Its subscales comprised of 27 items grouped into four factors: physical, functional, emotional, and social/family well-being. It is scored on a four-point Likert scale, with higher scores indicating better QOL. There is a Japanese version, and its reliability and validity have been confirmed.
(iii) Cancer-chemotherapy Concerns Rating Scale (CCRS): The CCRS was developed by Kanda [35]; its reliability and validity have been confirmed. This scale contains 15 items scored on a four-point Likert scale, from 1 to 4. It has four subscales (Self-existence, Disease progress, Reorganization of daily life, and Social and economic concerns) that are aligned with the four subconcepts. Its internal consistency and stability have been confirmed to be high, with a Cronbach’s α of .88.

These were all shown to demonstrate criterion-related validity.

#### Medical records and interviews

Data, including those of the diagnosis, medications, and doses taken were obtained from medical records. The investigators confirmed the presence of paresthesia of the hands or feet due to peripheral neuropathy (CTCAE, ver. 4) during examinations conducted, before asking the subjects to complete the questionnaire.

## Data analysis

IBM SPSS Statistics Ver.24 (SPSS Inc., Chicago, IL, USA) was used for all data analysis.

### Item analysis

Means and standard deviations (SD) were computed for each of the 40 items. Because all the questions were scored from 0 to 4, the settings were assigned such that the mean + 1 SD was ≥5 (ceiling effect) and mean – 1 SD was ≤0 (floor effect). Among items with a floor effect, those with a mean minus SD of > − 0.2 were retained, since one of the purposes was to screen for CAS-CIPN symptoms.

### Examination of validity types and reliability

1. Construct validity: Factor analysis was performed by maximum likelihood extraction using a promax rotation for the items. After the item analyses, the level at which an item was retained was set at a factor loading of > 0.4; for items with such factor loading, for several factors, only those with high clinical utility were retained. Oblique rotation was retained because correlations were assumed to exist between categories on the scale. A model was then produced for confirmatory factor analysis, and goodness of fit was confirmed by covariance structure analysis. Goodness of fit was evaluated by the goodness of fit index (GFI), adjusted goodness of fit index (AGFI), comparative fit index (CFI), and root mean square error of approximation (RMSEA).
2. Criterion-related validity: Criterion-related validity was examined by comparing participants’ CAS-CIPN scores with their GOG-Ntx scores, FACT-G scores, and CCRS scores.
3. Discriminant validity
  To examine discriminant validity, the participants with the highest 13% of GOG-Ntx scores were classed as the high-scoring group and those with the lowest 13% as the low-scoring group, and a t-test was used to investigate discriminative power from the total score on the scale.
4. Examination of reliability
  Cronbach’s α was obtained for the entire scale and for each subscale as an index of reliability. As peripheral neuropathy is dose-dependent, retesting was deemed to be unfeasible, and stability was instead investigated using the Spearman-Brown formula *q* = 2*r*/(1 + *r*).

#### Ethical considerations

The study protocol was reviewed and approved by the Ethics Committees of all the institutions from which data were collected. Ethical Committee For Clinical Studies, Gunma University Faculty of Medicine13–16 (include Red Cross Hospital Ethics and Hidaka Hospital Ethics:2013),The Ethics committee of The Jikei University School of Medicine for Biomedical Research 25–290(7425) and Niigata Prefectural Central Hospital Ethics Review Committee 2013–12. The participants were provided with oral and written explanations of the purpose of the study, what was required of them, the time required, and how their rights would be protected. Written informed consent was obtained prior to their participation in the study.

## Results

Valid responses were obtained from 327 of 358 individuals who agreed to participate in the study (valid response rate 91.3%), with 31 excluded because of inadequate responses.

### Participant attributes

The participants comprised 129 men (39.4%) and 198 women (60.6%). Their ages ranged from 25 to 89 years, with a mean of 61.0 (SD ± 11.5) years. There were 141 participants aged 60 years or younger (14.5%), while 186 participants were aged 61 years or older (56.9%). Gastrointestinal cancer, breast cancer, and cancer of the female reproductive organs, in that order, were the most common diagnoses. By CIPN CTCAE grade, 36.7, 57.8, and 5.5% were grades 1, 2, and 3, respectively. Regarding the location of paresthesia, 13.8, 10.1, and 76.4% involved the hands only, feet only, and both hands and feet, respectively. The drug most often used was paclitaxel in 44.6% of cases, followed by vincristine (Table 1).

**Table 1:** Participant Characteristics *n* = 327

Parameter	Breakdown	n	%
Sex	Male	129	39.4
	Female	198	60.6
Age	≦60	141	43.1
	61≦	186	56.9
Employment status	Unemployed	87	26.6
	Full time employee	84	25.7
	Homemaker	76	23.2
	Part time employee	40	12.2
	Retired	40	12.2
Performance status	0	78	23.9
	1	218	66.7
	2	31	9.5
Tumor location	Gastrointestinal organs	121	37.0
	Breast	79	24.2
	Female reproductive organs	47	14.4
	Blood/hematopoietic system	40	12.2
	Respiratory organs	15	4.6
	Other/unknown	25	7.6
Stage	I	25	7.6
	II	54	16.5
	III	86	26.3
	IV	160	48.9
	Unknown	2	0.6
CTCAE Peripheral sensory neuropathy	Grade 1	120	36.7
	Grade 2	189	57.8
	Grade 3	18	5.5
Paresthesia of the hands and feet	hands	45	13.8
	feet	33	10.1
	Both hands and feet	249	76.1
Drug used	Paclitaxel	146	44.6
	Oxaliplatin	85	26.0
	Vincristine	33	10.1
	Docetaxel	29	8.9
	Cisplatin	11	3.4
	Carboplatin	2	0.6
	Vinblastine	2	0.6
	Other	19	5.8

*CTCAE* Common Terminology Criteria for Adverse Events

### Item analysis

One item in the final version that exhibited a ceiling effect and 10 that exhibited a floor effect were excluded. Pearson’s product-moment correlation coefficient was calculated, and seven items that exhibited a somewhat strong correlation (*r* > 0.65) as well as four items with a weak correlation with the total score (*r* < 0.30) on I-T correlation analysis were excluded.

### Exploratory factor analysis and factor naming (Table 2)

Exploratory factor analysis of the 19 factors remaining after item analysis was performed by the principal factor method and promax rotation. Using the principal factor method, the Kaiser-Meyer-Olkin measure of sampling validity was 0.821 (Bartlett’s sphericity test *p* < 0.001), demonstrating its validity. On a scree plot, the slope was high between 4 and 5, and four factors had eigenvalues of ≥1. In promax rotation analysis, convergence was achieved after five iterations, and no item had factor loading of ≤0.4. However, several factors displayed high factor loading, and four were excluded for this reason. When the same analysis was repeated with 15 factors, no item failed to meet the criteria, and convergence was reached with four factors comprising 15 items (Table 2). These constituted the final CAS-CIPN. The cumulative contribution ratio was 64.58%.

**Table 2:**
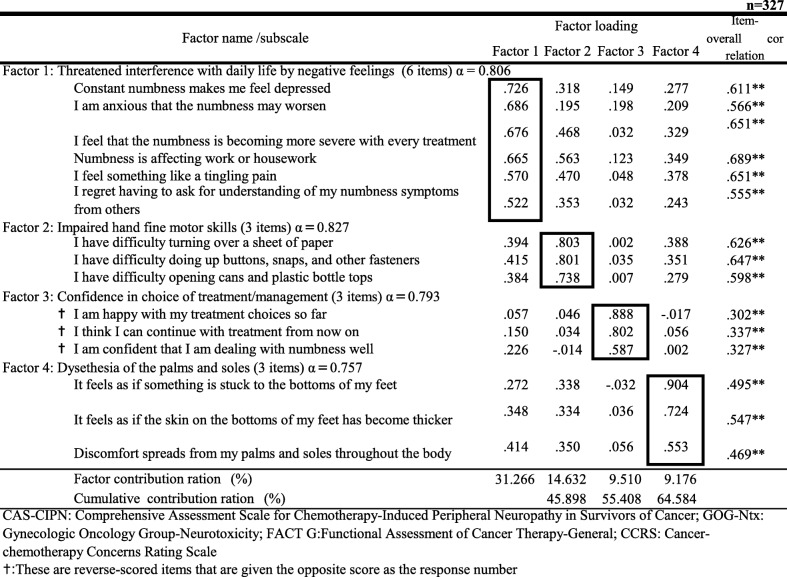
Results of the factor analysis *n* = 327

*CAS-CIPN* Comprehensive Assessment Scale for Chemotherapy-Induced Peripheral Neuropathy in Survivors of Cancer, *GOG-Ntx* Gynecologic Oncology Group-Neurotoxicity, *FACT G* Functional Assessment of Cancer Therapy-General, *CCRS* Cancer-chemotherapy Concerns Rating Scale **p < .01

✝:These are reverse-scored items that are given the opposite score as the response number

These factors were interpreted and named as follows: Factor 1 is concerned with the effect of negative feelings (such as depression and anxiety) on work and/or housework, and was named *Threatened interference in daily life by negative feelings*. Factor 2, with the inability to carry out fine manipulation with the hands, and was named *Impaired hand fine motor skills.* Factor 3, with the effect of treatment choice and management, and was named *Confidence in the choice of treatment/management*, and Factor 4 is concerned with perceptual disturbances in the hands and feet and was named *Dysesthesia of the palms and soles*.

### Confirmatory factor analysis

A hypothetical model was produced with hypothetical interfactor covariance between the 4 factors and 15 items obtained from the exploratory factor analysis. The goodness of fit indices were GFI = 0.885, AGFI = 0.862, CFI = 0.883, and RMSEA = 0.079, just failing to meet the criteria for significance, but the path coefficients were all significant at ≥0.5 (*p* < 0.01). Because the study participants were selected based on having already experienced chronic peripheral neuropathy, this may have been a biased sample. On the basis of an investigation of these findings, it was judged that they did not contradict the exploratory factor analysis.

### Investigation of reliability

Cronbach’s α was 0.826 for the entire scale, 0.860 for *Threatened interference in daily life by negative feelings*, 0.826 for *Impaired hand fine motor skills*, 0.793 for *Confidence in choice of treatment/management*, and 0.757 for *Dysesthesia of the palms and soles.* All these exceeded the criterion for reliability of 0.70, confirming the internal consistency of the entire scale and its subordinate factors. The factors and items that were finally included were numbered in order, starting from Factor 1, and split-half analysis was carried out with the items divided into odd-numbered and even-numbered questions. The coefficient of reliability calculated using the Spearman-Brown formula was 0.713 (*p* < 0.01), confirming the stability of the scale.

### Investigation of validity

#### Criterion-related validity

**Fig. 2:**
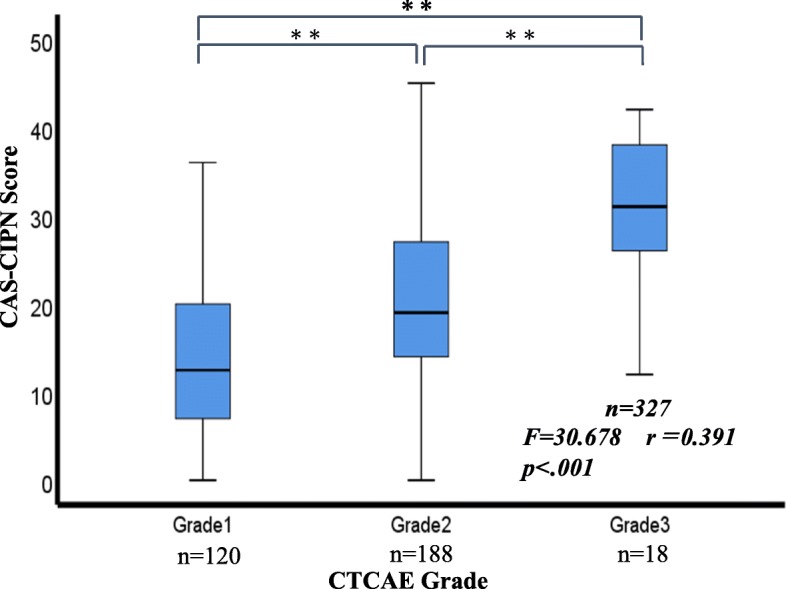
The relationship patient-reported CAS-CIPN and nurse- reported CTCAE (Peripheral sensory neuropathy). **Significant difference between groups (*p* < 0.01). CAS-CIPN: Comprehensive Assessment Scale for Chemotherapy-Induced Peripheral Neuropathy in Survivors of Cancer; CTCAE: Common Terminology Criteria for Adverse Events

To investigate the association between this scale and external criteria, its correlations with the GOG-Ntx, FACT-G, and CCRS were investigated. The correlation coefficient between the GOG-Ntx and the CAS-CIPN developed in this study was 0.714 (*p* < 0.01). The correlation coefficient with the FACT-G was *r* = − 0.403 (*p* < 0.01) and that with the CCRS was 0.452 (*p* < 0.01), both indicating moderate correlations (Tables 3 and 4). Moreover, in the correlation coefficients between CAS-CIPN score and CCRS subscale score, only factor 2 and “Social and economic concerns” did not exhibit correlations. Figure 2 shows the relationship between patient-reported CAS-CIPN and nurse-reported CTCAE (peripheral sensory neuropathy). For grade 1, CAS-CIPN scores were distributed from 0 to 40 with a mean of 13.8 (SD 8.8), for grade 2, scores were distributed from 0 to 47 with a mean of 20.1 (SD 10.08), and for grade 3, scores were distributed from 12 to 42 with a mean of 20.1 (SD 10.08). The F value was 30.488 (*p* < 0.0001), with significant differences also observed in later testing. Further, r = 0.391 (*p* < 0.0001), indicating a significant correlation. Criterion-related validity was thus confirmed by these correlations with the GOG-Ntx, FACT-G, and CCRS.

**Table 3:** Correlation coefficient between CAS-CIPN and GOG-Ntx, Total FACT-G and FACT-G Subscale Score *n* = 327

	FACT-GOG-Ntx Score	Total FACT-G Score	FACT-G Subscale			
			Physical	Functional	Emotional	Social/family
CAS-CIPN	.714**	−0.403**	−.443**	−.263**	−.333**	−0.013
Factor 1 score	.614**	−0.401**	−.500**	−.221**	−.382**	0.035
Factor 2 score	.653**	−0.143**	−.260**	− 0.042	−.196**	0.073
Factor 3 score	0.038	−0.326**	−0.070	−.395**	− 0.044	−.232**
Factor 4 score	.559**	−0.184**	−.254**	−0.078	−.194**	0.030

*CAS-CIPN* Comprehensive Assessment Scale for Chemotherapy-Induced Peripheral Neuropathy in Survivors of Cancer, *GOG-Ntx* Gynecologic Oncology Group-Neurotoxicity, *FACT G* Functional Assessment of Cancer Therapy-General

**p < .01

**Table 4:** Correlation coefficient between CAS-CIPN Score and Total CCRS Score, CCRS Subscale Score *n* = 327

	Total CCRS Score	The CCRS Subscale			
		Self-existence	Disease progress	Reorganization of daily life	Social and economic concerns
CAS-PCIPN	.452**	.418**	.338**	.317**	.303**
Factor 1 score	.456**	.405**	.372**	.297**	.321**
Factor 2 score	.190**	.197**	.121*	.180**	0.076
Factor 3 score	.252**	.215**	.196**	.170**	.192**
Factor 4 score	.285**	.273**	.181**	.207**	.201**

*CAS-CIPN* Comprehensive Assessment Scale for Chemotherapy-Induced Peripheral Neuropathy in Survivors of Cancer, *CCRS* Cancer-chemotherapy Concerns Rating Scale

* *p* < .05,**p < .01

#### Discriminative validity

Study participants were divided into a high-scoring group (*n* = 44, 13.6%) and a low-scoring group (*n* = 43, 13.1%) in terms of their total GOG-Ntx scores, and their total CAS-CIPN scores. The mean score for the high-scoring group was 31.2 points and that for the low-scoring group was 8.6 points, a significant difference of 22.6 points (*p* < 0.001), confirming the discriminative validity of the CAS-CIPN.

## Discussion

We confirmed that the CAS-CIPN is a comprehensive assessment scale with high reliability and validity. The CAS-CIPN has three distinctive features (the most outstanding of these is the 15 items, which provides a comprehensive measurement of the effects of CIPN) and four subscales (*Threatened interference in daily life by negative feelings*, *Impaired hand fine motor skills*, *Confidence in the choice of treatment/management*, and *Dysesthesia of the palms and soles*). These enable a comprehensive assessment of physical sensations and their effects on daily life, including the psychological (mental) aspects and social relationships, and how patients deal with these.

Two systematic reviews of existing CIPN assessment tools (Griffith et al. 2010 [18], Haryani et al. 2017 [15]) both recommended the GOG-Ntx and the TNS, which are widely used worldwide. The advantage of the TNS is that it combines subjective assessment by the patient with objective assessment by a medical professional. However, it is more complex to administer because it includes nerve conduction measurements, which require training to perform. Furthermore, medical professionals tend to underestimate the severity and frequency of CIPN, particularly subjective symptoms, which affect patients’ function and QOL [17, 19]; thus, it has been suggested that sensitive studies, focused on patients’ reports, may be required [4, 16].

The GOG-Ntx assesses the state of physical symptoms (physical sensations) and limitations on daily life, but does not measure its comprehensive effects, including psychological and mental aspects. The scale developed in this study included four more items than the GOG-Ntx, and covers the psychological and mental aspects and how the patients deal with them; making it a highly comprehensive scale that holds promise for clinical use. Because this scale is for use in patients expected to suffer from CIPN, the small number of items means that it will not impose a heavy burden on respondents. In this sense, 15 items are a tolerable number, ensuring patients participation and reducing the likelihood of missing values. This means that peripheral neuropathy can be assessed at an early stage, enabling intervention to prevent it from becoming severe.

The second feature is that its development was based on the experience of Japanese survivors; thus, all questions utilized a language that is easily understood by Japanese patients. Numerous survivors used the phrases: “Numbness is affecting work or housework” and “I feel that the numbness is becoming more severe with every treatment,” at the questionnaire development stage; and this content has been reflected in the CAS-CIPN.

The PNQ, which was also developed in Japan [21], distinguishes between the incidence and severity of sensory and motor impairment, and interference with activities of daily living. The self-check sheet [23] measures sensory and motor impairment and pain. However, neither of these includes items concerning chopstick use or kneeling, activities that are part of Japanese daily life. Further, it is unclear whether in the process of developing these scales if life activities reflecting Japan’s unique culture were included. While developing this scale, the idea of including life activities, which reflects the culture was raised, to address the goal of developing a comprehensive scale, but these items were removed after the pilot test. They were covered by the items “I feel something like a tingling pain,” “I have difficulty doing up buttons, snaps, and other fasteners,” and “ It feels as if the skin on the bottoms of my feet has become thicker”, which covers the physical sensations, hand fine motor skills, and dysesthesia of the palms and soles. This prevented the underestimation of symptoms that occur in patient-reported assessment tools, including items on physical sensations and activities of daily living [25, 31]. The PNQ is a sheet for monitoring the hands and feet when using taxane, platinum, and other drugs. Further, when oxaliplatin is added the details of a case can be understood through oral monitoring or other means. Like the PNQ, this scale captures the effects on the hands and feet. While it may not be an Asian-specific instrument, we believe it is capable of subjectively assessing CIPN.

The most prominent characteristic of this scale is that it not only examines the effects of CIPN on the body and on daily activities, but it is able to comprehensively assess the physical, emotional, social, spiritual, and behavioral aspects. In addition, as Fig. 1 shows, there was a significant correlation between patient-reported CAS-CIPN and nurse-reported CTCAE. Finally, while the PNQ may be a superior scale, ours is simple and can be filled out quickly at hospital outpatient centers and clinics, despite having a large number of items.

Medical treatment today emphasizes not only extending survival but also QOL. The questionnaire items “I am happy with my treatment choices so far,” “I think I can continue with treatment from now on,” and “I am confident that I am dealing with numbness well”, reflect a survivor’s self-efficacy, which serves to increase his or her capacity for self-management and independence. Bandura [36, 37] described a theory when people act at their level of capacity and with confidence in carrying out a task or behavior. Self-efficacy is important to increasing the self-care abilities of cancer survivors [38]. Even with CIPN, it is important to increase the capacity for self-care and raise level of confidence so that they can work and engage in safe and preventative behavior. For this reason, it is an essential part of our scale.

The third feature is the positive correlation between CAS-CIPN and GOG-Ntx. This supports the conceptual model used in this study. In the development of the CAS-CIPN, the following four subconcepts were theoretically established: physical sensations, effect on daily life, effect on relationships and social roles, and mental/psychological/spiritual effects. However, factor analysis resulted in the identification of four factors: *Threatened interference in daily life by negative feelings*, *Impaired hand fine motor skills*, *Confidence in choice of treatment/management*, and *Dysesthesia of the palms and soles*. Of those, *Impaired hand fine motor skills* and *Dysesthesia of the palms and soles* (physical sensation) corresponded to the theory. *Threatened interference in daily life by negative feelings* had a complex association with physical, psychological/mental, social, and spiritual aspects, symbolized by expressions such as “Constant numbness makes me feel depressed” and “I regret having to ask for understanding on my numbness symptoms from others.” Score on Factor 3 of the CAS-CIPN, *Confidence in choice of treatment/management*, was only weakly correlated with GOG-Ntx score, and this was a new factor that was identified in this study. Both the last two factors represent concepts not included in existing multidimensional scales, and are distinctive features of the CAS-CIPN as a scale comprehensively assessing the effects of CIPN.

## Limitations of the study

There are three limitations to this research. The first is that the study participants were limited to Japanese. It is necessary to prove within an expanded scope that CIPN is applicable to cancer survivors in Asia and around the world, to enhance the generalizability. Second, this survey was subjective evaluation only. In future, it is necessary to perform both objective and subjective evaluations simultaneously, to improve the accuracy, based on the relationship between the two evaluation approaches. Furthermore, the participants in the study were selected because they had CIPN, and this may have resulted in bias.

## Conclusions

We developed the CAS-CIPN using 327 cancer survivors with PCIPN who had undergone at least 6 courses of regimens using taxane or other drugs that caused CIPN, and had continued on these therapies. We demonstrated that the CAS-CIPN had high reliability and validity as a scale for comprehensively assessing the symptoms of CIPN and their effects. It exhibited high internal consistency and stability, with Cronbach’s α 0.826 and coefficient of reliability 0.713. CAS-CIPN scores were strongly correlated with those of the GOG-Ntx (*r =* 0.714, *p* < 0.01), confirming both the criterion-related validity and the discriminative validity of this scale.

The CAS-CIPN consists of 15 items and is easy to use. It enables cancer survivors to provide medical professionals with information on their CIPN in an effective manner, and offers valuable information for the self-management of CIPN. In the future, we hope to examine how this scale correlates with objective indices such as the TNSc score and PNQ, as well as to examine its applicability in cancer patients. Longitudinal studies are essential to investigate the value of the CAS-CIPN in future.

Further studies are required to determine whether or not this scale is also easy to use in other countries.

## Data Availability

All data supporting the findings are included in this publication.

## Abbreviations

CAS-CIPN: The Comprehensive Assessment Scale for Chemotherapy-induced peripheral neuropathy
CCRS: Cancer-chemotherapy Concerns Rating Scale
CIPN: Chemotherapy-induced peripheral neuropathy
CTCAE: Common Terminology Criteria for Adverse Events
FACT-G: Functional Assessment of Cancer Therapy-General
GOG-Ntx: The Functional Assessment of Cancer Therapy/Gynecologic Oncology Group-Neurotoxicity
PCIPN: Persistent CIPN
PNQ: Patient Neurotoxicity Questionnaire
PS: Performance Status
QLQ-CIPN20: Quality of life questionnaire- Chemotherapy-induced peripheral neuropathy
QOL: Quality of life
SD: Standard deviations
